# Data of 16S rRNA gene amplicon-based metagenomic signatures of arecanut rhizosphere soils in Yellow Leaf Disease (YLD) endemic region of India

**DOI:** 10.1016/j.dib.2021.107443

**Published:** 2021-10-05

**Authors:** S. Paulraj, Ravi Bhat, M.K. Rajesh, S.V. Ramesh, U.K. Priya, R. Thava Prakasa Pandian, Vinayaka Hegde, P. Chowdappa

**Affiliations:** ICAR-Central Plantation Crops Research Institute, Kasaragod, Kerala 671124, India

**Keywords:** Arecanut, Metagenome amplicon sequencing, Rhizosphere, Yellow Leaf Disease

## Abstract

Arecanut (*Areca catechu* L.) is an important plantation crop cultivated predominantly in the Indian states of Karnataka, Kerala, Assam, West Bengal, and Maharashtra in an area of 5.19 lakh ha, with Karnataka State alone accounting for about 68.41% of the area and 79.97% of production. Arecanut production has recently been hampered due to environmental and disease pressures, especially the escalating incidence of Yellow Leaf Disease (YLD). The involvement of phytoplasma as the etiological agent of YLD has been reported. Symptoms include yellowing at the tip of leaflets of two or three fronds of the outer most whorl which gradually spreads to the inner whorl of leaves. As the disease progresses, the entire crown becomes yellow leaving only the spear leaf green. In severe cases, the affected leaves often show necrosis from their tips. In advanced stages, the leaves are reduced in size and become stiff and pointed and the crown ultimately falls off. Degeneration of cortex is commonly observed in the diseased roots. The kernel of affected nuts shows discolouration and later turns blackish. The reduction in yield over a period of three years, immediately after the incidence of the disease, has been estimated to be around 50%. Harnessing the arecanut–microbiome interactions to address the biotic and abiotic stresses of the host plant offers immense opportunity to increase arecanut production sustainably. Here, we report a comprehensive analysis of the structural composition of the arecanut rhizosphere bacterial diversity utilizing next-generation sequencing (NGS) technology. We have used amplicon sequencing (V3-V4 regions of the 16S rRNA gene) of bulk soil and rhizosphere samples collected from YLD endemic regions of Aranthodu, Sullia Taluk, Dakshina Kannada District, Karnataka State, India, to assess the microbial diversity. The results revealed that while there is a great diversity of bacterial communities, relatively few bacterial phyla predominate with higher relative abundance. The phyla *viz*., Proteobacteria, Bacteroidetes, Firmicutes, Acidobacteria, Planctomycetes, Patescibacteria, Chloroflexi, Actinobacteria, Fusobacteria, and Verrucomicrobia were found to be dominant in the rhizosphere of the arecanut.

## Specifications Table

**Table:** 

Subject	Agriculture and Biological Sciences
Specific subject area	Metagenomics
Type of data	Metagenome amplicon sequence data, tables, figures, text files
How data were acquired	Illumina MiSeq platform
Data format	Raw, filtered, analyzed
Parameters for data collection	Amplicon sequencing (V3-V4 regions of the 16S rRNA gene) of bulk soil and rhizosphere samples collected from arecanut fields from YLD disease-endemic regions of Aranthodu, Sullia Taluk, Dakshina Kannada District, Karnataka State, India.
Description of data collection	1. Collection of soil samples from the rhizosphere of healthy palms (YLD-AHR), diseased palms (YLD-DIR) and non-rhizosphere region (YLD-NR) during the monsoon season 2. Isolation of DNA from rhizosphere/non-rhizosphere soil using QIAamp® DNA microbiome Kit 3. Amplification of V3-V4 regions of the 16S rRNA gene using KAPA HiFi HotStart Ready Mix, sequencing on Illumina MiSeq platform and data analysis
Data source location	Aranthodu, Sullia Taluk, Dakshina Kannada District, Karnataka State, India.
Data accessibility	Repository name: NCBI SRA Data identification number: Bio-project-PRJNA721704 (Accession Nos: SRR14252056 to SRR14252061) Direct URL to data: https://www.ncbi.nlm.nih.gov/sra/PRJNA721704

## Value of the Data

• The data generated provides baseline information regarding the bacterial communities of the arecanut rhizosphere in YLD endemic regions of Aranthodu, Sullia Taluk, Dakshina Kannada District, Karnataka State, India, recorded during the peak symptomatic, monsoon period.
• The data provides information about the natural distribution of different bacterial species in the arecanut rhizosphere community. This will be used as a baseline for further investigating the spatial and temporal shift in the microbial community under different growing conditions and/ or biotic/abiotic stresses.
• The results provide invaluable information to researchers to guide and harness the potential and sustainable microbial inoculants, technology to improve plant production and health in arecanut plantations in the YLD endemic regions.

## 1 Data Description

**Fig. 1:**
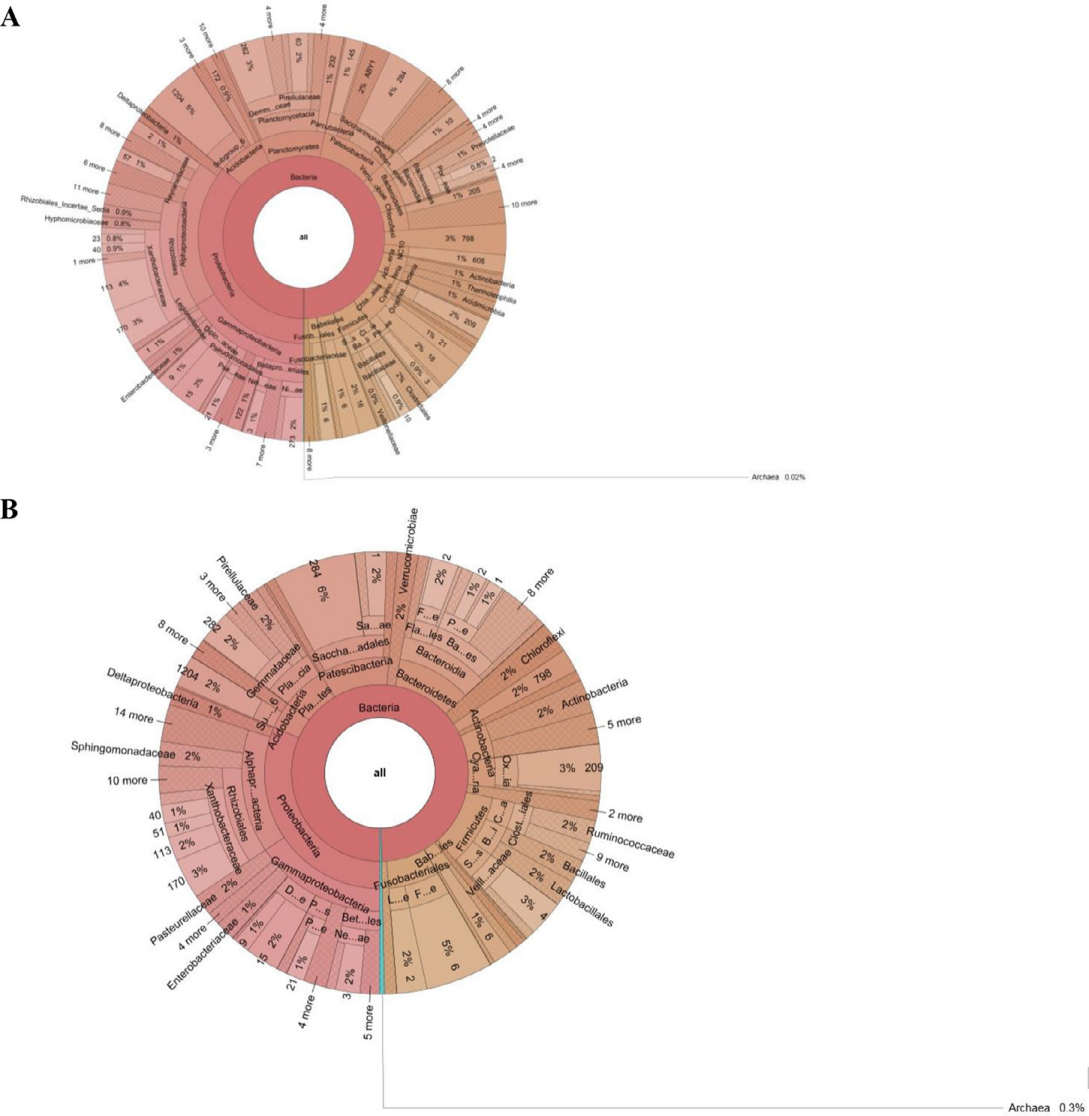

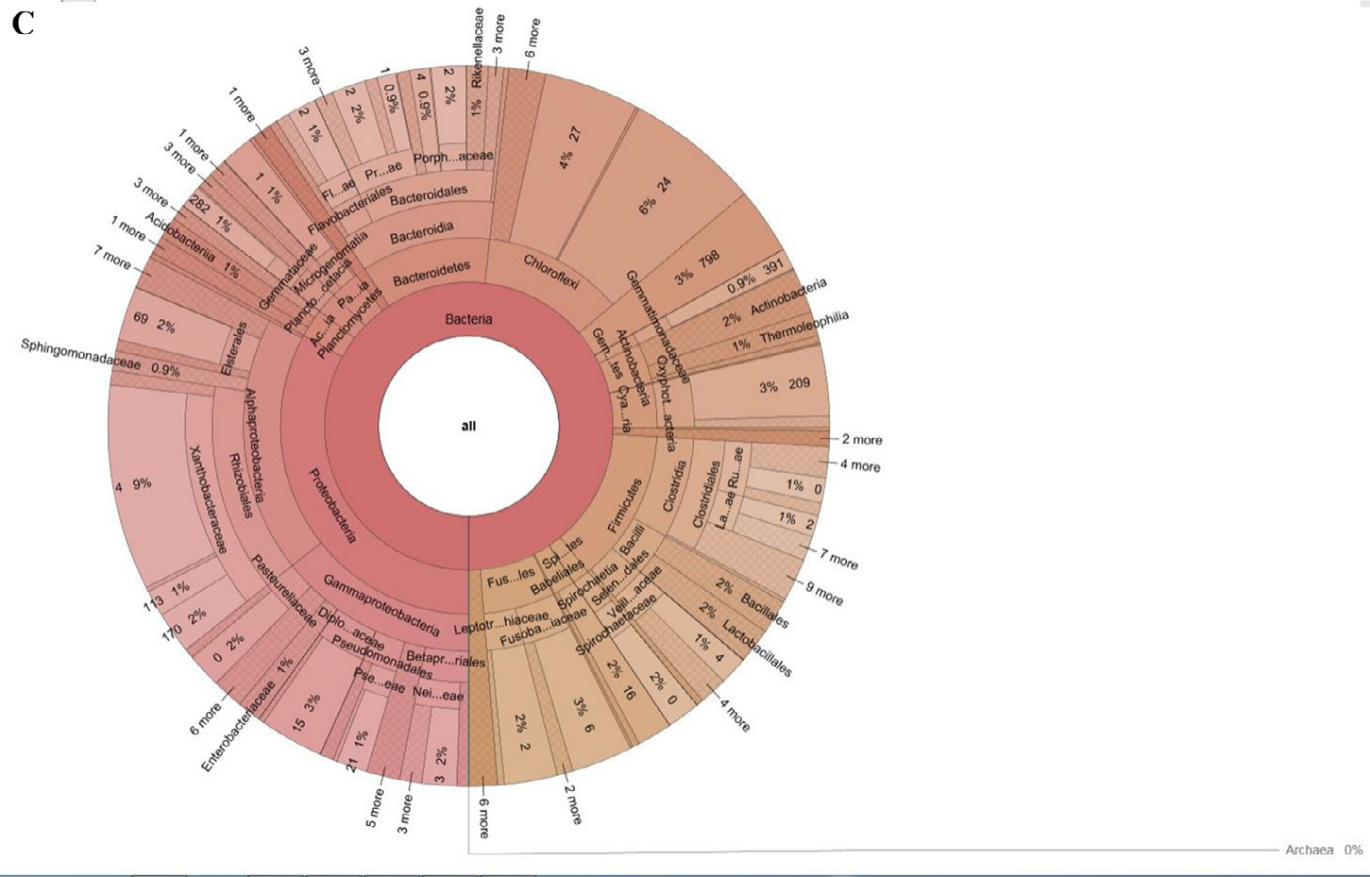
Distribution of various bacterial communities in the arecanut YLD endemic rhizosphere soil in Aranthodu-Sullia (Krona Pie Chart reflecting the distribution of microbiome). **A** YLD endemic Apparent Healthy Rhizosphere soil (YLD-AHR). (**B**) YLD endemic Disease Intensive Rhizosphere soil (YLD-DIR). (**C**) YLD endemic Non-Rhizosphere soil (YLD-NR).

**Fig. 2:**
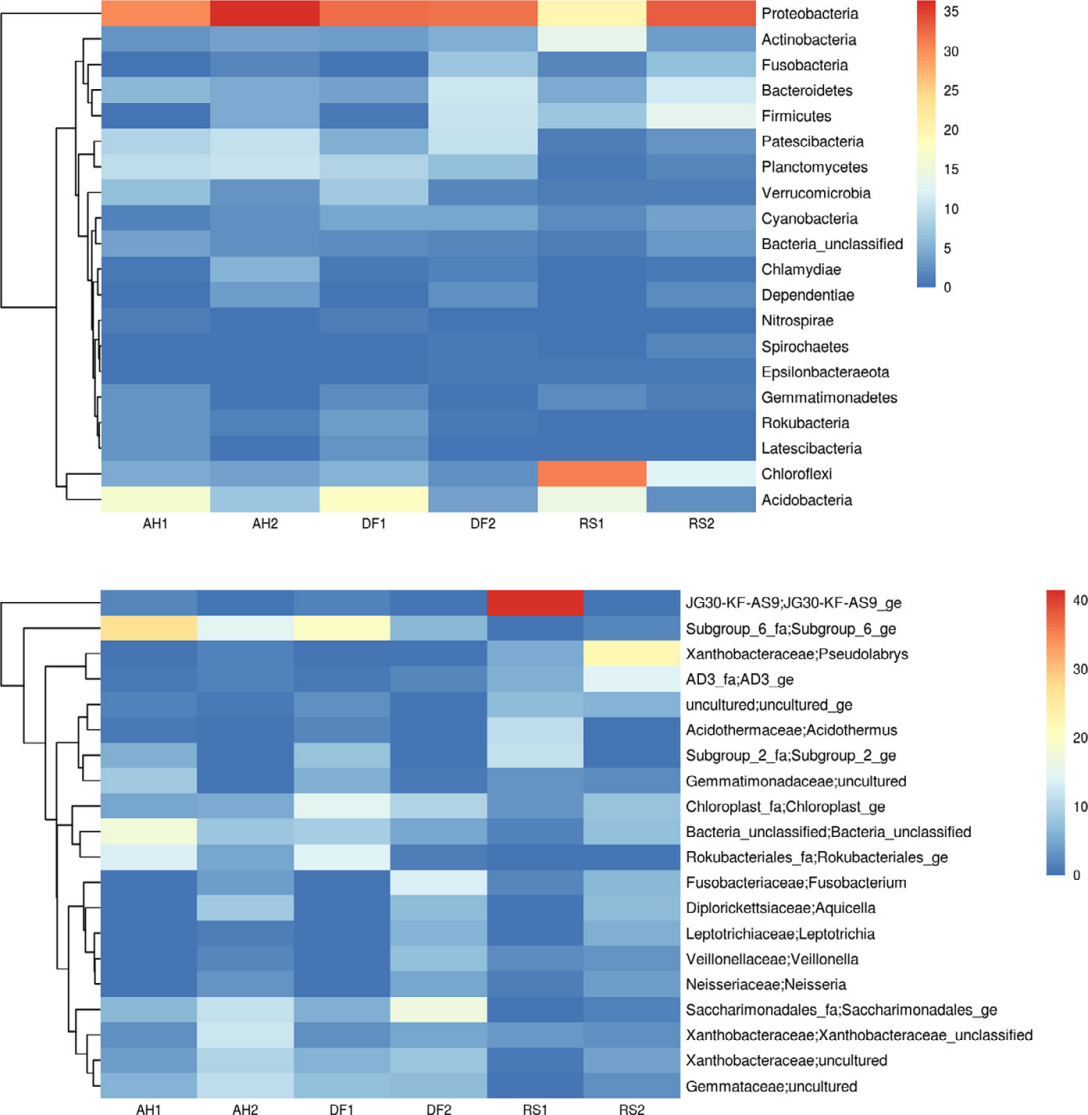
**A** Phylogenetic tree of predominant Phyla OTUs and their heat map in YLD endemic rhizosphere soil in Aranthodu, Sullia. **B** Phylogenetic tree of predominant Genera OTUs and their heat map in YLD endemic rhizosphere soil in Aranthodu, Sullia.

The dataset describes the predominant bacterial communities present in the arecanut rhizosphere obtained using amplicon sequencing (V3-V4 regions of the 16S rRNA gene) of bulk soil and rhizosphere samples collected from YLD endemic regions of Aranthodu, Sullia Taluk, Dakshina Kannada District, Karnataka State, India. The details of the samples collected, the number of reads and the quality of reads obtained on an Illumina MiSeq platform of rhizosphere soil from the YLD endemic area of in Aranthodu are provided in Supplementary File S1 and Supplementary File S2. The compositions of various bacterial communities in the arecanut YLD endemic rhizosphere are presented in Figs. 1 and 2. The contig sizes, number of Operational Taxonomic Units (OTUs), pooled abundance and relative abundance of the predominant Phyla in rhizosphere soils of apparent healthy rhizosphere soil (YLD-AHR); YLD endemic disease intensive rhizosphere soil (YLD-DIR); YLD endemic non-rhizosphere soil (YLD-NR) are given in Supplementary File S3.

A total of 632,304 paired-end raw reads, with a sequence length cut off 300 bp, were acquired through sequencing. Taken together, a total of 181,289 reads were obtained after sequencing with a flattering rarefaction curve (Supplementary File S4), which yielded 91396 contigs corresponding to 1174 OTUs of bacterial genera belonging to 47 OTUs of bacterial phyla from soils of the YLD endemic area. A total of 58,935 contigs corresponding to 937 OTUs of bacterial genera were obtained in YLD-AHR samples, 55,666 contigs aligning to 836 OTUs of bacterial genera in YLD-DIR samples, and 66,688 contigs corresponding to 452 OTUs for YLD-NR soil samples.

## 2 Experimental Design, Materials and Methods

### 2.1 Collection of rhizosphere soil samples

The rhizosphere soil samples were collected from YLD endemic Apparent Healthy Rhizosphere soil (YLD-AHR); YLD endemic Disease Intensive (YLD-DIR); YLD endemic Non Rhizosphere soil (YLD-NR) from Aranthodu, Sullia Taluk, Karnataka State, India. Palms of about 30–40 years of South Kanara Local cultivar, predominantly grown in that area, were selected. Samples were collected from the active root zones, 30–45 cm away from the trunk core and at 5–40 cm depth, where the root system was denser from the surface. The samples were processed and stored in refrigerated conditions for further downstream analysis. Samples were collected from three palms per site, and three samples were collected from each palm and pooled to avoid sampling variations.

### 2.2 DNA extraction

The sampled roots with rhizosphere soil particles attached were placed in sterile tubes containing 9 mL of physiological solution (9 g/L NaCl). The tubes were vortexed for 5 min to detach the soil particles and then centrifuged at 4000 rpm for 5 min. The supernatant was discarded, and the remaining soil fraction was used for DNA extraction using the metagenomic soil DNA extraction kit (QIAamp®). DNA extraction was done in three replications, and the corresponding samples were pooled (Supplementary File S5)

### 2.3 Library preparation

The V3-V4 regions of the 16S rRNA gene were amplified using the 341F and 785R primers [1]. The amplicons were purified, and adapters were added to sequence the libraries. Library preparation was done and quantified using the fluorometric method [2]. The denatured library was then subjected to paired-end sequencing on an Illumina MiSeq platform.

### 2.4 Data analysis

The data quality of the raw reads was checked by FastQC [3] and MultiQC [4]. The reads were trimmed (20 bp) from the 5’ end to remove the degenerate primers. The trimmed reads were processed to remove adapter sequences and low-quality bases using Trimgalore [5]. The QC passed reads were imported into Mothur [6], and the pairs were aligned to form contigs. The contigs were screened for errors, and only those between 300 bp and 532 bp were retained. Any contig with ambiguous base calls was rejected. The high-quality contigs were checked for identical sequences, and the duplicates were merged. After this process, the gaps and the overhang at the ends of the contigs were removed and processed for chimera removal, which might have formed due to errors in PCR conditions. UCHIME algorithm [7] was used to flag contigs with chimeric regions. The filtered contigs were processed and classified and were clustered to the Operational Taxonomic Units (OTUs). Sequences were binned to OTUs at 97% sequence similarity with USEARCH [8] using an agglomerative clustering algorithm. Then, a representative sequence of each OTU was further used to estimate the bacterial diversity using the Metagenomics Rapid Annotation pipeline (Silva v.132 database) [9] to obtain the taxonomical diversity of bacteria and archaea. Proteobacteria, Bacteroidetes; Firmicutes; Acidobacteria, Planctomycetes, Patescibacteria, Chloroflexi, Actinobacteria, Fusobacteria and Verrucomicrobia were the dominant phyla in the rhizosphere of the arecanut palms.

## Ethics Statement

Not applicable.

## Data Availability

The supplementary files for this article are curated in the data repository as below:

PAULRAJ, SANTHAPPAN (2021), “Data of metagenomic signatures of arecanut rhizosphere soils in Yellow Leaf Disease (YLD) endemic region”, Mendeley Data, V1, https://doi.org/10.17632/5zp738z82z.1

## CRediT authorship contribution statement

**S. Paulraj:** Conceptualization, Data curation, Formal analysis, Writing – original draft. **Ravi Bhat:** Supervision, Funding acquisition. **M.K. Rajesh:** Conceptualization, Writing – review & editing. **S.V. Ramesh:** Conceptualization, Writing – review & editing. **U.K. Priya:** Methodology, Resources. **R. Thava Prakasa Pandian:** Methodology, Resources. **Vinayaka Hegde:** Supervision, Funding acquisition. **P. Chowdappa:** Supervision, Funding acquisition.

## Declaration of Competing Interest

The authors declare that they have no known competing financial interests or personal relationships that could have appeared to influence the work reported in this paper.

## Appendix Supplementary materials

**Supplementary File S1.** YLD endemic arecanut rhizosphere soil in Aranthodu-Sullia sample, location details, metagenomic DNA quality and number of reads and quality of reads obtained by Illumina Miseq sequencing and the sequence accession numbers in repository. Image, application 1

**Supplementary File S2.** Arecanut palm showing characteristic symptoms associated with Yellow Leaf Disease (YLD) Image, image 2

**Supplementary File S3.** The contig size, number of OTUs, pooled abundance and relative abundance of the predominant Phyla in Apparent Healthy Rhizosphere soil (YLD-AHR); YLD endemic Disease Intensive Rhizosphere soil (YLD-DIR); YLD endemic Non-Rhizosphere soil (YLD-NR). Image, application 3

**Supplementary File S4**. Rarefaction curve shows the measure of diversity that has been captured by a given number of reads in samples of YLD endemic arecanut rhizosphere soil in Aranthodu-Sullia Image, image 4

**Supplementary File S5**. Genomic DNA gel image of YLD endemic rhizosphere soils Image, image 5
