# Supplementary figures and images for: Data of 16S rRNA gene amplicon-based metagenomic signatures of arecanut rhizosphere soils in Yellow Leaf Disease (YLD) endemic region of India
Source: Data Brief. 2021 Oct 5;38:107443. doi: 10.1016/j.dib.2021.107443 (PMC8551408; doi:10.1016/j.dib.2021.107443)

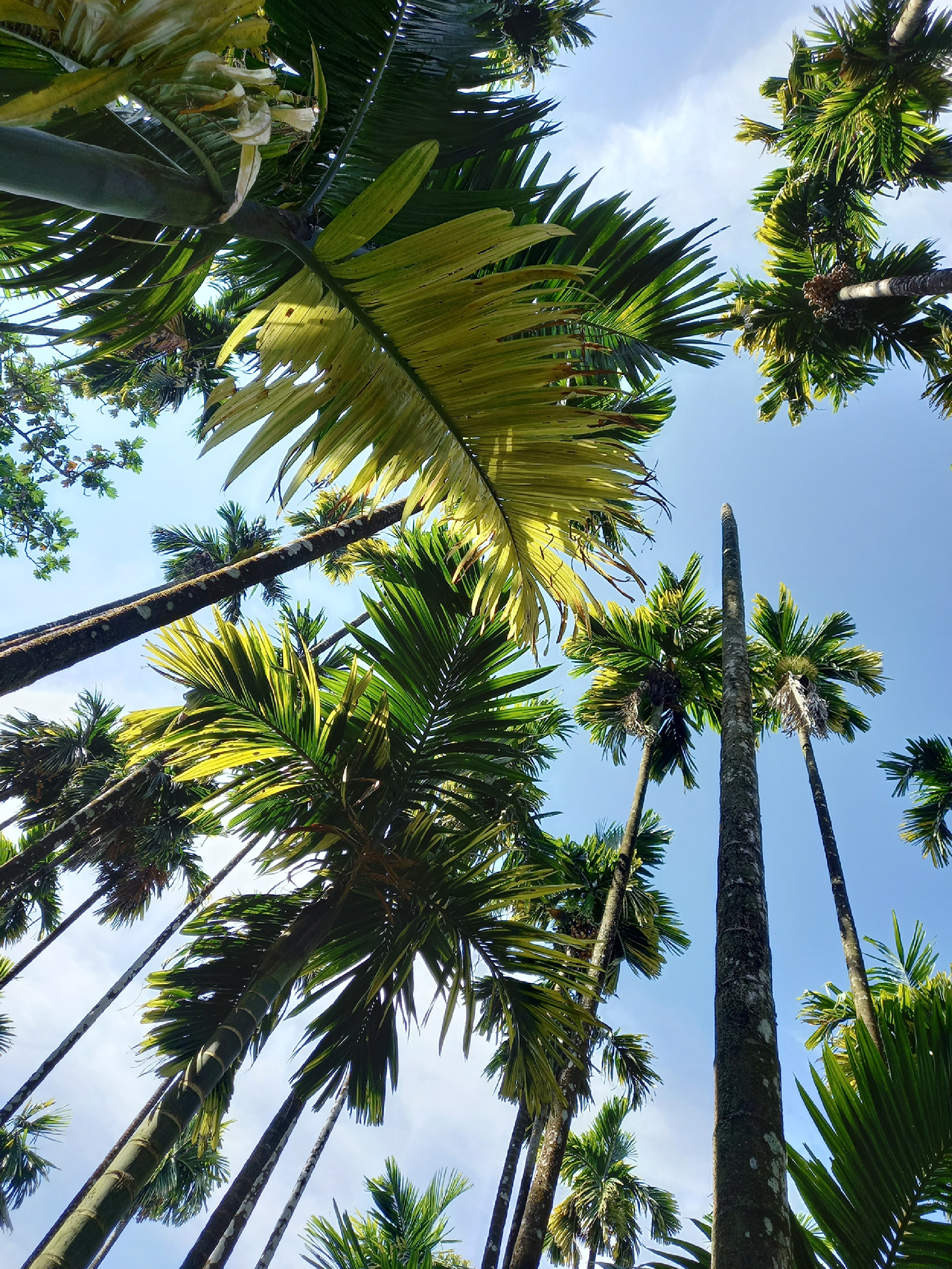

Supplement: Supplementary file 2 — Supplementary File S2. Arecanut palm showing characteristic symptoms associated with Yellow Leaf Disease (YLD) [file mmc2.jpg]

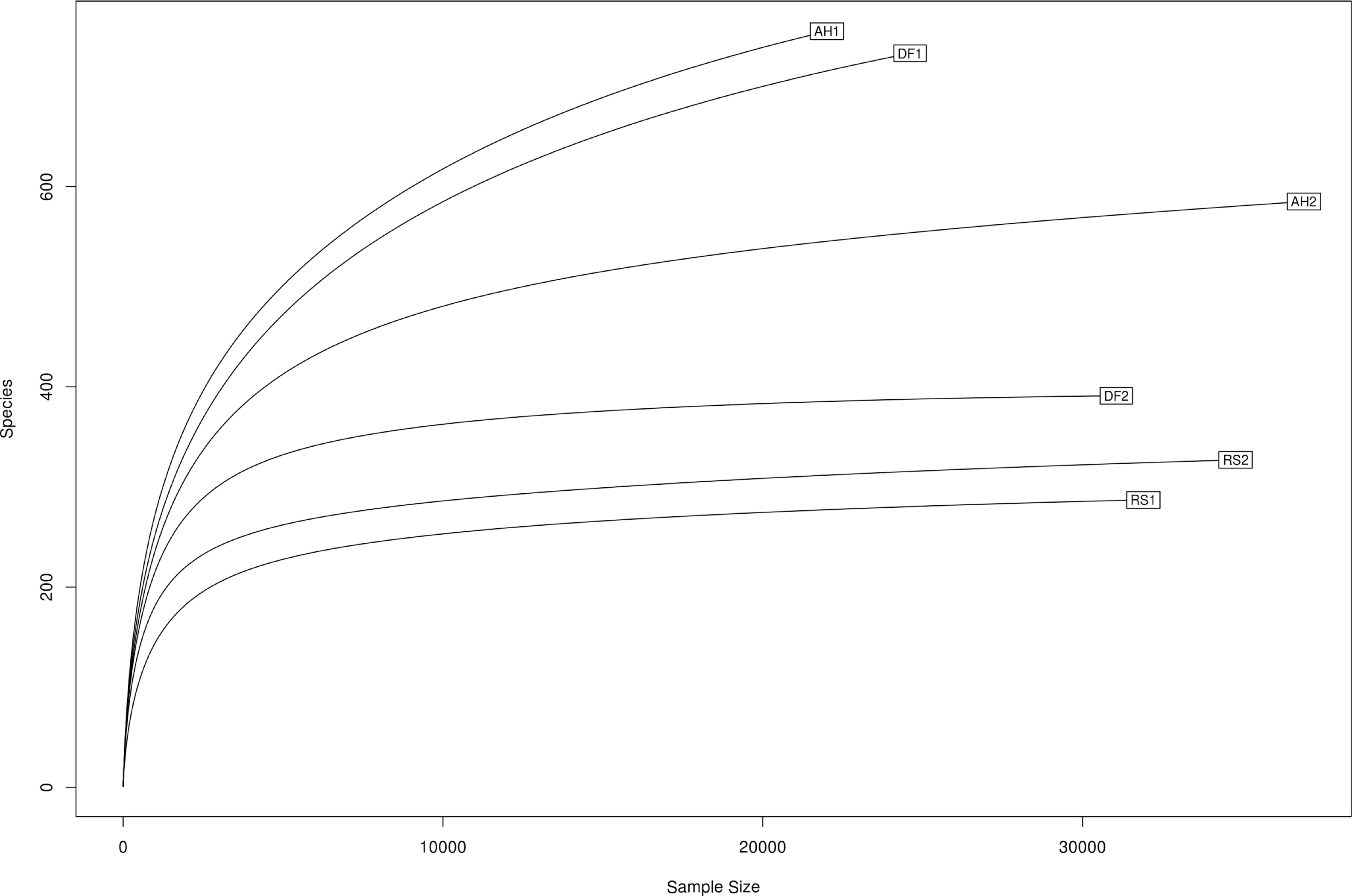

Supplement: Supplementary file 4 — Supplementary File S4. Rarefaction curve shows the measure of diversity that has been captured by a given number of reads in samples of YLD endemic arecanut rhizosphere soil in Aranthodu-Sullia [file mmc4.jpg]

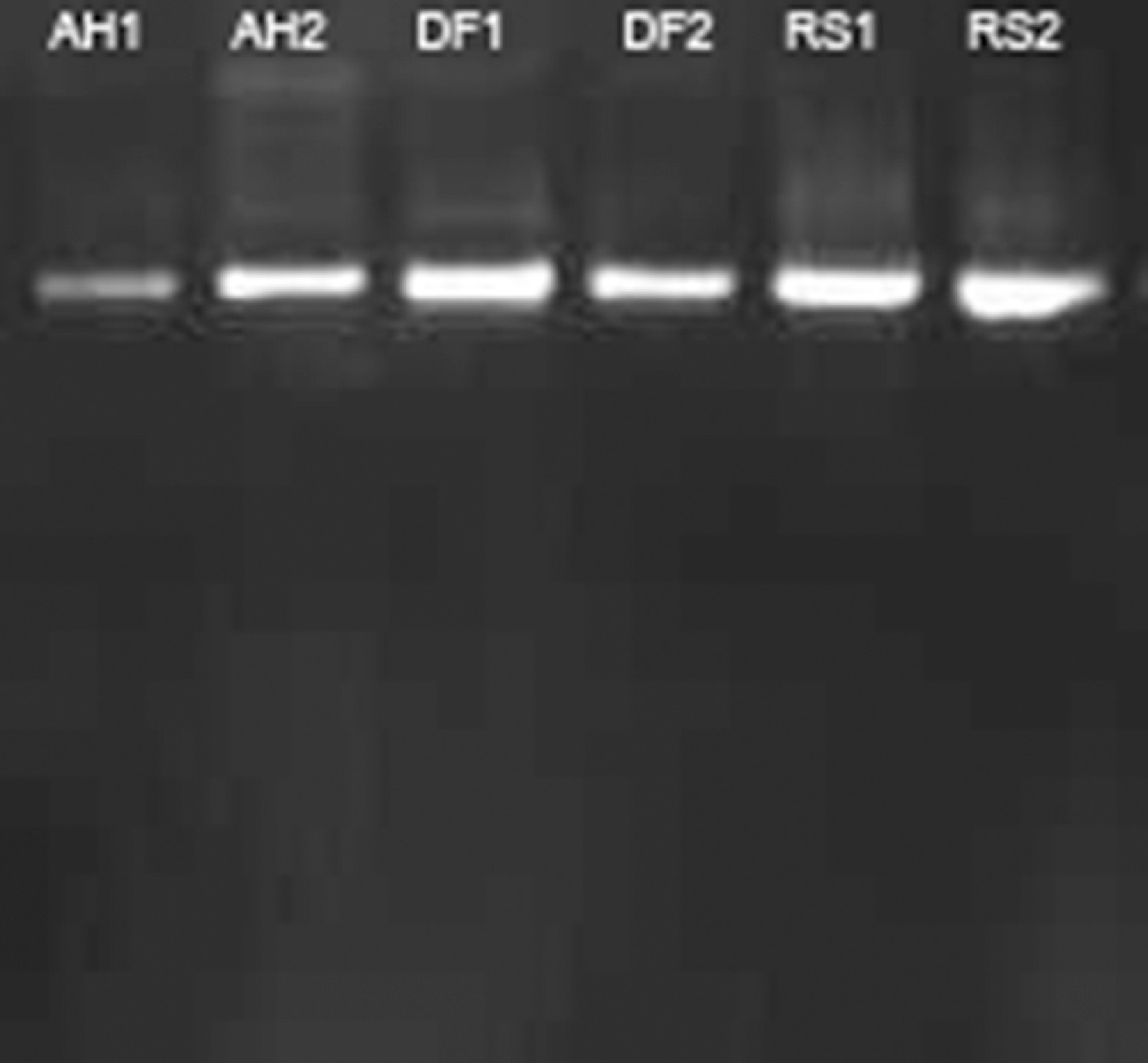

Supplement: Supplementary file 5 — Supplementary File S5. Genomic DNA gel image of YLD endemic rhizosphere soils [file mmc5.jpg]
